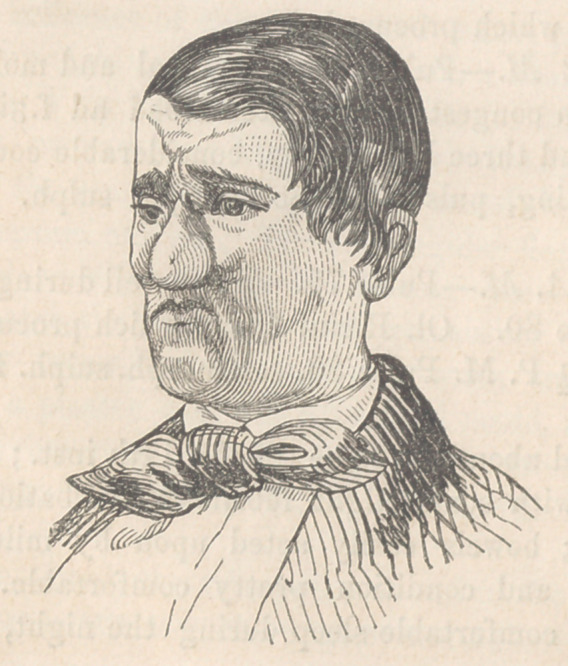# Cases of Plastic Surgery, by Prof. Gilbert

**Published:** 1851-04

**Authors:** H. J. Richards

**Affiliations:** Clerk of the Medical and Surgical Dispensary


*Cases of Plastic Surgery, by Prof.* GILBERT. *Clinique of the Pennsylvania College.* Reported by Dr. H. J. Richards, Clerk of the Medical and Surgical Dispensary.

From our register of surgical cases requiring operative proceedings, I select the two following, as possessing in themselves peculiar interest; the first demonstrating the occasional success attending operations performed for the restoration of parts destroyed by malignant disease; the second presenting some modifications in procedure, rendered necessary by the extent of the injury, and the nature of the structure which remained.

*Case* 1.—Miss Sarah Queer, æt. 36, residence, Lehigh Co., Pa. Presented for operation by her medical attendants, Drs. Detweiler and Martin, who stated that the patient had lost her nose by lupus, and that the parts had cicatrized, and continued well for four years. On examination, all the cartilages, more than half of the ossa nasi, vomer, the whole of the spongy portion of the ethmoid, and the contiguous portion of the ossa maxillæ superioris, were found destroyed. The site of the organ was occupied by a cicatrix, in the centre of which was a small circular opening about three-eighths of an inch in diameter, around which the integument was drawn into radiating plicæ.

The patient being in good health and condition, the operation was performed in the presence of the class of the College, on the afternoon of the same day, (Nov. 24th, 1849,) in the usual manner, by bringing a flap of integument from the forehead, after having made preparations for its reception by incisions down to the bone, through the margins of the opening, between the nasal processes of the superior maxilla. The integuments and cicatrix included within these incisions, were separated above and laterally, to be used in the formation of the septum in the construction of the new nose. The patient was rendered insensible to pain by the inhalation of a mixture of chloroform and ether. Under appropriate after treatment the case progressed favorably for about ten days; quick union took place between the flap and its new attachment. At this juncture, erysipelatous inflammation made its appearance, in consequence of which all treatment, except that demanded by this new state of things, was suspended. The patient having recovered, it was found that the septum was entirely destroyed, leaving, however, the union that had already taken place, unimpaired. Fearing the return of the erysipelatous inflammation, it was thought advisable not to perform the supplementary operations for the restoration of the septum, or the division of the pedicle during that session.

*Oct.* 21*st*, 1850. *Supplementary Operations in the above Case.*

*Remarks.*—You recognize the subject of a plastic operation performed in your presence last session. You see her in good health, and anxious for the remaining operations. The pedicle you perceive has lost its twist; the probe, however, passes readily between it and the part subjacent. It will be sufficient therefore, merely to freshen the edges, and make incisions into the skin adjacent, and then to attach the margin of each side of the pedicle to the skin, without disturbing the circulation. As to the septum, you perceive that granulations have been thrown out, so as to occlude entirely the nasal cavities. Nothing remains, therefore, but to puncture the solid structure on each side of the mesial line, in the course of the natural apertures, so as to make a communication with the general cavity of the nares; and to keep it patent by means of glass tubes, until the surface is covered with mucous membrane.

These operations were accordingly performed, and the patient presents the highly improved appearance represented in cut 2, from a daguerreotype by Laughlin.

*Case* 2. *December* 7*th*, 1850. H. Hullinger, æt. 21, residence, Gettysburg, Pa. The hideous aspect of the subject of this operation, suggested the forbidding spectacle of the dissecting table—a human face without a countenance—no feature remaining in its integrity, to impart a living expression to the scarred remains. The following cuts from daguerrotypes taken prior to the operation, convey in some degree, the appearance of the unfortunate patient.

This frightful mutilation was the result of a gun-shot wound. The circumstance occurred about 13 years ago, as follows. Being at play in a room with a companion, who was amusing himself with a loaded musket, the piece was accidentally discharged, the whole contents, consisting of an ounce ball and three buckshot with wadding, passed through the face of the patient, entering at the right side, and causing the most extensive destruction of the soft parts of the face, anywhere to be found in the records of surgery. Nearly every bone between the lower jaw and the cranium, was either displaced, or shattered. The nasal processes of the superior maxilla, turbinated bones, vomer, and ossa lachrymalia, were entirely removed, or discharged during the process of healing. The anterior portion of the os ethmoides, as well as the anterior parts of the ossa maxillæ sup., were also carried away. The remaining fragments of the latter bones, with the ossa palati, were disarticulated, and fell together, so as to obliterate the nasal passages. The highmorean cavities were exposed, and there remained only enough of the alveolar margins to contain five molar teeth, which, owing to the loss of intervening substance, approximated, so as to place them considerably posterior to, and consequently within the incisors of the lower jaws. The destruction of soft parts was equally extensive. The buccinator region of both sides, the upper lip, and portions of the masseter muscles were removed. The integument below the eyelids to the extent of about an inch on the right, and rather less upon the left side, with part of the nasal integument, only remained intact. The parts were adjusted after the accident, without any expectation that recovery was possible. The patient, after a tedious confinement of several months, survived, and has since enjoyed excellent health. His habits are good, and at this time his system is in the best possible condition for a successful operation. This, and his urgent desire for an operation, remarked Dr. Gilbert, have induced me to make an effort to improve his appearance by means of plastic surgery, with the aid of anæsthesia. The destruction of bone and soft parts having been so extensive, as you perceive, it would be a boast to promise more than an improvement. The depression in the centre of his face, the large amount of cicatrix, the consequent scarcity of material, from which to re-construct an upper lip, all present difficulties not met with in ordinary rhinoplastic operations. It is my design to bring the material for the nose from the forehead, except the septum, which can be brought up from the remains of the nasal integument; and to construct the lip by means of two rectangular flaps from the cheeks, drawn together so as to unite at the mesial line.

The usual preparations having been made, the operation was accordingly performed in the presence of the class, by Dr. Gilbert, assisted by Drs. Darrach, Grant and Hunter. When completely under the influence of chloroform and ether, proceedings were commenced by making incisions to include a triangular flap upon the forehead. V incisions were then made for its reception in its new position, preparations having been previously made for the attachment of the remaining fragment of nasal integument, intended for the septum narium. The rectangular flaps, designed to supply the place of the upper lip having been separated from the cheeks, and the cicatrices and remaining soft parts dissected from the bones beneath, an impression in wax was taken, from which a plaster cast was afterward made, representing the shallow highmorean cavities, and the relative position of the superior maxillæ and teeth, to the lower maxillary bone.

The incisions completed, the triangular wound in the forehead was closed by suture connecting the angles, and adhesive strips, with lint dressing. The flap was twisted on its pedicle, and secured in the grooves for its reception by three double sutures on each side. These sutures were introduced by using double armed ligatures, each extremity being first passed through the integument on the inner side of the sulcus, next through the margin of flap, and lastly brought out through the outer margin of sulcus, thus dovetailing, as it were, the margin of the flap into the corresponding incisions made for its reception. The flaps for the new lip were retained in apposition by two large gold needles and one of smaller size in front; and secured above by stitches of interrupted suture to parts prepared for their attachment. The open spaces on the cheeks were secured as on the forehead, and dressed with lint. Supplementary operations were required to repair the want of union at different points, owing to the feeble organization of the large amount of old cicatrix, which constitutes the material. Operation from 9½ to 10¾ A. M.

I shall abbreviate the daily report made by the senior students who had charge of the case from date of the operation until 16th inst., after which time his condition required no special attention.

*Dec. 7th, 5 P. M.*—No collapse, pulse 68, skin in good condition, patient comfortable. 8½ P. M. Pulse 72, quick. Dr. Gilbert punctured the nose to relieve congestion. Sol. morph. sulph. f. ʒiss., which procured sleep.

*Dec. 8th, 12 M.*—Pulse 72, skin cool and moist. 8 P. M. Pulse 78, organ congested, evacuated blood ad f.ʒiii. 12, midnight. Has had three hours sleep, considerable cough and dyspnœa on awaking, pulse 80. Sol. morph. sulph. Had an inefficient stool.

*Dec. 9th, 7 A. M.*—Pulse 75. Slept well during the morning. 2 P. M. Pulse 80. Ol. Ricini f.℥ss., which procured two good evacuations. 9½ P. M. Pulse 88, sol. morph. sulph. f.ʒiss. et pulv. Ipecac gr. ii.

He continued about the same till the 11th inst.; pulse averaging about 90, with some slight febrile exacerbation and cough towards night; bowels easily acted upon by mild purgatives; skin pleasant, and condition pretty comfortable. Upon the 11th, after a comfortable sleep during the night, he awoke at

7 A. M., with quickness and frequency of pulse. Several stitches were removed; a purgative enema exhibited, as also sol. morph. sulph. and aq. lauro-cerasi. 9 A. M. Pulse reduced to 100; delirium gone.

The patient progressed through the remainder of his convalescence, without any notable symptoms; requiring simply an antiphlogistic and sedative treatment with simple dressings. Satisfactory union took place between all essential points, excepting the anterior margin of the two rectangular flaps, which rendered a supplementary operation for simple hare-lip necessary.

Having completely regained his strength, this, as well as the usual operation upon the pedicle was performed. The former was sufficiently united at the 5th day to allow of the removal of the needles. The latter was performed as in our first case, the object being here to elevate the bridge by permitting the twist to remain. Dr. Gilbert stated that in those cases where the pedicle was divided, and communication through that channel thereby cut off, he was in the habit of making compression upon it for a few weeks prior to the operation, in order that the collateral circulation might be fairly established.

The following cut is from daguerreotype taken eleven weeks after the operation.

## Figures and Tables

**Figure f1:**
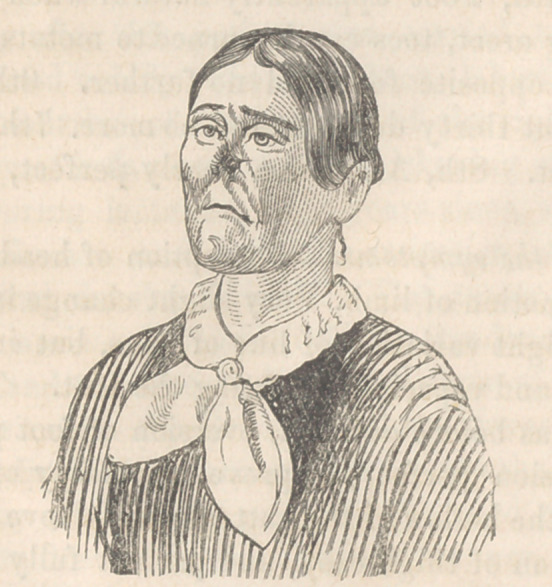


**Figure f2:**
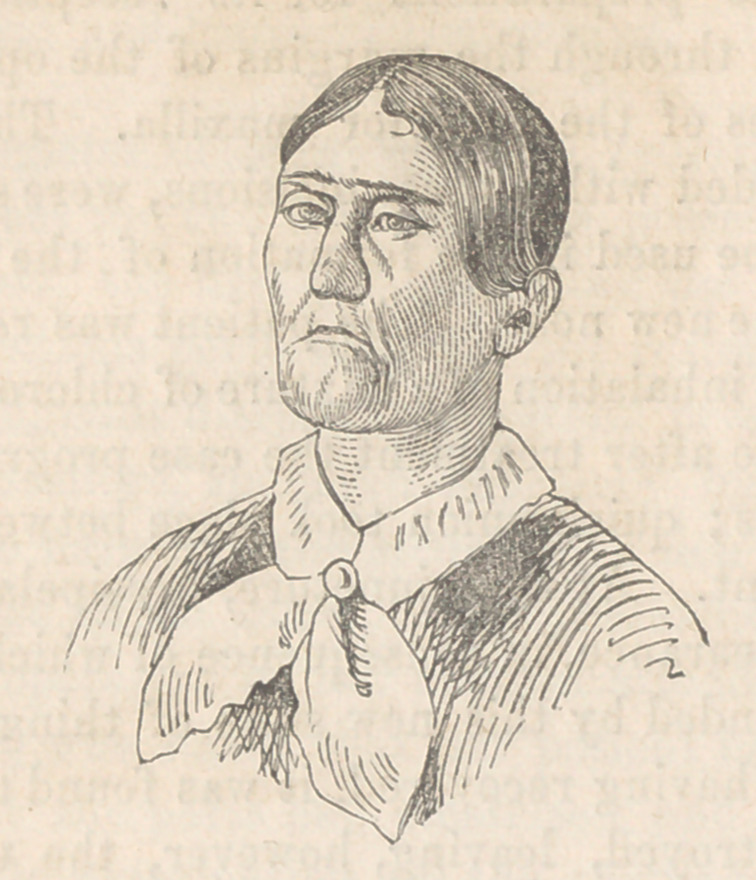


**Figure f3:**
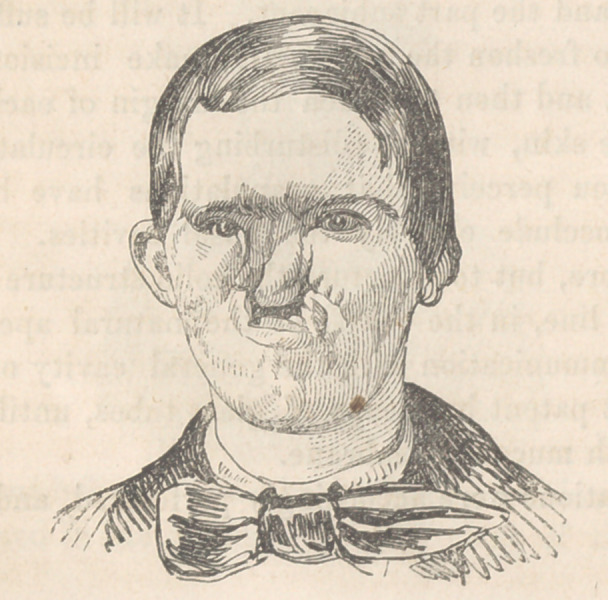


**Figure f4:**
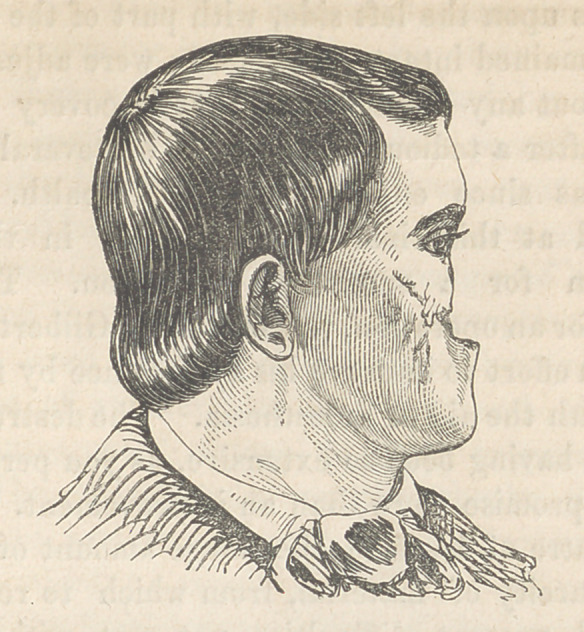


**Figure f5:**